# A Critical Role for the mTORC2 Pathway in Lung Fibrosis

**DOI:** 10.1371/journal.pone.0106155

**Published:** 2014-08-27

**Authors:** Wenteh Chang, Ke Wei, Lawrence Ho, Gerald J. Berry, Susan S. Jacobs, Cheryl H. Chang, Glenn D. Rosen

**Affiliations:** 1 Division of Pulmonary and Critical Care Medicine, Stanford University School of Medicine, Stanford, California, United States of America; 2 Division of Pulmonary and Critical Care Medicine, University of Washington School of Medicine, Seattle, Washington, United States of America; 3 Department of Pathology, Stanford University School of Medicine, Stanford, California, United States of America; University of Pittsburgh, United States of America

## Abstract

A characteristic of dysregulated wound healing in IPF is fibroblastic-mediated damage to lung epithelial cells within fibroblastic foci. In these foci, TGF-β and other growth factors activate fibroblasts that secrete growth factors and matrix regulatory proteins, which activate a fibrotic cascade. Our studies and those of others have revealed that Akt is activated in IPF fibroblasts and it mediates the activation by TGF-β of pro-fibrotic pathways. Recent studies show that mTORC2, a component of the mTOR pathway, mediates the activation of Akt. In this study we set out to determine if blocking mTORC2 with MLN0128, an active site dual mTOR inhibitor, which blocks both mTORC1 and mTORC2, inhibits lung fibrosis. We examined the effect of MLN0128 on TGF-β-mediated induction of stromal proteins in IPF lung fibroblasts; also, we looked at its effect on TGF-β-mediated epithelial injury using a Transwell co-culture system. Additionally, we assessed MLN0128 in the murine bleomycin lung model. We found that TGF-β induces the Rictor component of mTORC2 in IPF lung fibroblasts, which led to Akt activation, and that MLN0128 exhibited potent anti-fibrotic activity in vitro and in vivo. Also, we observed that Rictor induction is Akt-mediated. MLN0128 displays multiple anti-fibrotic and lung epithelial-protective activities; it (1) inhibited the expression of pro-fibrotic matrix-regulatory proteins in TGF-β-stimulated IPF fibroblasts; (2) inhibited fibrosis in a murine bleomycin lung model; and (3) protected lung epithelial cells from injury caused by TGF-β-stimulated IPF fibroblasts. Our findings support a role for mTORC2 in the pathogenesis of lung fibrosis and for the potential of active site mTOR inhibitors in the treatment of IPF and other fibrotic lung diseases.

## Introduction

Idiopathic Pulmonary Fibrosis (IPF) is a devastating disease, which afflicts over 200,000 patients in the United States and Europe [1]. The pathogenesis is unknown but a dysregulated wound healing response to lung epithelial injury, which leads to progressive interstitial fibrosis, is a hallmark of the disease. Activated fibroblasts in fibroblastic foci secrete a variety of pro-fibrotic proteins in response to TGF-β, such as type I and type III collagen, fibronectin (FN), and the matricellular family members, secreted protein acidic and rich in cysteine (SPARC) and connected tissue growth factor (CTGF) [2].

The evolutionary conserved serine/threonine protein kinase mTOR is a member of the phosphatidylinositol 3-kinase (PI3K)-related kinase (PIKK) family [3]. mTOR integrates both extracellular and intracellular signals and acts as a central regulator of cell metabolism, growth, proliferation and survival [4]. In mammalian cells, mTOR resides in two physically and functionally distinct signaling complexes: mTOR complex 1 (mTORC1), a rapamycin-sensitive complex, and mTOR complex 2 (mTORC2) [5], [6]. The mTORC1 complex consists of at least five components: (i) mTOR, the catalytic subunit of the complex; (ii) Raptor; (iii) mLS8; (iv) PRAS40; and (v) Deptor; mTORC1 phosphorylates the ribosomal S6K1 (protein S6 kinase 1) and 4E-BP1 (eukaryotic translation initiation factor eIF4E binding protein 1) proteins, which regulate growth and protein synthesis, respectively [7]. Rapamycin and related rapalogs are known allosteric inhibitors of mTORC1 but do not generally directly inhibit mTORC2, although prolonged treatment with rapamycin suppresses mTORC2 in some cell types [8]. Also, the inhibition of mTORC1 by rapamycin can activate mTORC2 and thereby activate Akt [9]. A recent study showed that rapamycin failed in an IPF clinical trial [10].

The mTORC2 complex consists of six different known proteins: (i) mTOR; (ii) Rictor; (iii) mSIN1; (iv) Protor-1; (v) mLST8; and (vi) Deptor. Rictor and mSIN1 mutually stabilize each other, thus establishing the structural foundation of the complex [7]. The mTORC2 complex mediates the phosphorylation of Akt on Ser473 and thereby activates the downstream Akt pathway, which regulates multiple cellular responses, including increased cell growth and proliferation, a shift to glycolytic metabolism, and increased cell migration [11]. In response to growth factors, PI3K stimulates phosphorylation of Akt at Thr308 through activation of phosphoinositide-dependent protein kinase 1 (PDK1) [11]. We showed previously that SPARC produced by IPF fibroblasts activates Akt by phosphorylation of serine 473 (Ser473) leading to inhibition of glycogen synthase kinase 3β (GSK-3β), which resulted in activation of the β-catenin pathway and inhibition of apoptosis [12]. Other studies have shown that loss of phosphate and tensin homolog (PTEN) in IPF fibroblasts also causes activation of Akt, through phosphorylation at Ser473 [13], [14]. We hypothesized, therefore, that Akt activation in IPF lung fibroblasts is mediated by the mTORC2 component of the mTOR pathway.

The discovery of active site ATP-competitive mTORC1/2 inhibitors was recently reported by several research groups, although a selective mTORC2 inhibitor has yet to be developed. Several active site mTOR inhibitors, that block both mTORC1 and mTORC2, such as MLN0128 (previously known as INK128), have progressed to clinical trials for cancer [5], [15]–[17]. In this study, we show that the Rictor component of mTORC2 is induced by TGF-β in lPF lung fibroblasts, which was coincident with Akt activation. Also, we show that the active site mTOR inhibitor MLN0128 exhibits several properties, which suggest it may have antifibrotic activity in a clinical setting: (i) it inhibits expression of stromal proteins by IPF fibroblasts; (ii) it inhibits lung injury and fibrosis in a murine bleomycin model, and (iii) it protects lung epithelial cells from TGF-β-induced toxicity originating from IPF fibroblasts. These data suggest a role for mTORC2 as a mediator of lung fibrosis and suggest that active site mTOR inhibitors may hold promise for the treatment of fibrotic disease.

## Materials and Methods

### Ethics Statement

Informed consent was obtained with a Stanford IRB-approved protocol to obtain explant lung tissue from patients undergoing surgical lung biopsy for the diagnosis of an idiopathic interstitial pneumonia or lung transplant for IPF. Fibroblasts were isolated from the surgical lung explants.

All mice used in this research project are maintained in two animal rooms in the Division of Laboratory Animal Medicine. All mice are maintained under filter-top, barrier isolation and all cages are changed in a laminar flow hood. Critically important strains are maintained in rooms in which the cages, filter tops, bedding and food are autoclaved. At the present time, the mice are free of all known murine viruses and free of ecto- and endoparasites. Experimental mice are monitored on a daily basis for morbidity and are sacrificed if there is evidence of suffering. The colony as a whole are monitored every 2–3 months for the presence of antibodies to a standard panel of murine viruses, cultured for the presence of pathogenic bacteria and examined for parasites regularly. Every effort is made to ensure that the animals do not suffer any discomfort, distress, pain or injury beyond what is unavoidable in the conduct of this research. Animals that are part of a treatment group are evaluated on a daily basis for evidence of morbidity and are sacrificed if there is any appearance of suffering. Mice that are to be sacrificed for specific studies are euthanized by CO_2_ inhalation. Following any surgical procedures, the animals are warmed on a heating pad until they are awake and ambulating. All of the methods of euthanasia and anesthesia are consistent with the recommendations of the Panel of Euthanasia of the American Veterinary Medical Association.

#### Cells and reagents

Reagents were from Sigma-Aldrich (St. Louis, MO) or otherwise indicated. Lung fibroblasts were isolated from IPF patients obtained from surgical lung biopsy or lung transplant and cultured in DMEM/10% fetal bovine serum (Invitrogen, Grand Island, NY) as previously described [12]. All protocols were approved by Stanford Institutional Review Board and Administrative Panel on Biosafety. Cells were starved in 0.1% serum medium for 24 hours, before TGF-β (5 ng/ml) stimulation. A549 and RLE-6TN cells were from the American Type Culture Collection (Manassas, VA) and maintained following supplier’s instructions. PP242 and MLN0128 were from Chemdea (Ridgewood, NJ), and Takeda Pharmaceuticals (Deerfield, IL), respectively.

#### Western blot analysis

Western blot analysis was described previously [18] with following antibodies; type I collagen (Millipore, Billerica, MA), EDA-fibronectin (MP Biochemicals, Aurora, OH), α-SMA (American Research Products, Belmont, MA), SPARC (Biodesign International, Saco, ME), p-Akt (Ser473 or Thr308), Akt, p-S6, p-Smad2, p-Smad3, Raptor (Cell Signaling Technology, Danvers, MA), Smad2/3 (BD Biosciences, Franklin Lakes, NJ), Smad3 (Zymed, Life Technologies, Grand Island, NY), Smad4, Rictor (Santa Cruz Biotechnology, Dallas, Texas), Smad7 (Imgenex/Novus, Littleton, CO) and α-tubulin (Calbiochem/Millipore, Billerica, MA).

#### RNA interference

Constructs of raptor and rictor shRNA were from Addgene (plasmids 1857 and 1853, respectively) [11]. The SPARC shRNA, scramble, cell transduction, and selection procedures were described previously [12].

#### Bleomycin lung model

The murine bleomycin lung toxicity model was used as described previously [19]. Mice received intratracheal bleomycin (MP Biomedicals, Santa Ana, CA) at 1.0 U/kg body weight. Mice were treated by intraperitioneal delivery of vehicle (40% PEG400) or MLN0128 (0.75 mg/kg body weight) daily (6/7 days) starting at Day 0 (prevention model) or Day 7 (therapeutic model) after bleomycin. For the prevention model, three mice were used in saline, or MLN0128 groups, and six mice were used in bleomycin, or bleomycin+MLN0128 groups. Body weight was measured at day −1 (receiving treatment), day 0 (receiving bleomycin), day 4, 7, 11, and 14 when all surviving animals were collected from four independent experiments. For the therapeutic model, three mice were used in saline, or MLN0128 groups, six mice were used in each bleomycin group, and five mice were used in each bleomycin+MLN0128 group. Body weight for the therapeutic model was collected at day 0 (receiving bleomycin), day 3, 7 (the first day receiving treatment), 10, 14, 17, and day 21 when all surviving animals were collected from five independent experiments. One mouse from the bleomycin group was harvested at day 7 from each experiment to access lung histology prior to MLN0128 treatment. For the Sircoll collagen assay and Ashcroft analysis, data from surviving mice is combined from experiments, which are described above.

#### Histological analysis

The mouse left lung was assessed for fibrosis by the Ashcroft scale [20] as previously described [19].

#### Sircoll collagen assay

Collagen content of the right lung was determined per the manufacturer’s instructions (Biocolor Ltd., UK). In the prevention model, 2/3 of mice were used for the Sircoll collagen assay and 1/3 for gene expression analysis.

#### Transwell culture

Fibroblasts (before passage 8) were seeded in a 24-well plate at 5×10^4^ cells/well. After starvation, cells were pre-treated with inhibitors for 30 minutes before TGF-β treatment for 16 hours. A549 or RLE-6TN cells were plated at 1×10^4^ cells per transwell (BD Biosciences, Franklin Lakes, NJ), and starved for 24 hours. Treated-fibroblasts were washed twice with PBS and placed in starvation media before the insertion of epithelia-containing transwells. After a 48 hour incubation, the epithelia-containing transwells were transferred into new vessels and the viability of epithelia was determined by Alamar blue assay [21].

#### Measurement of H_2_O_2_ release

H_2_O_2_ release was measured through the conversion of Amplex Red reagent by peroxidase to produce the red-fluorescent oxidation product, resorufin [22]. Following treatment, IPF fibroblasts were washed twice, and incubated with a reaction mixture (100 µM Amplex red [Cayman Chemical, Ann Arbor, MI], 5 U/ml horseradish peroxidase, 1 mM HEPES in Hank’s Balanced Salt Solution without phenol red). After a 90 minute incubation, signals were measured with excitation and emission wavelengths at 544 and 590 nm, respectively. H_2_O_2_ concentrations were calculated by plotting against a standard curve.

#### Statistical Analysis

Results are expressed as mean ± standard deviation (SD). One-way analysis of variance and the Student’s t Test were used for inter-group comparison. A probability level of p<0.05 was considered significant.

## Results

**Figure 1:**
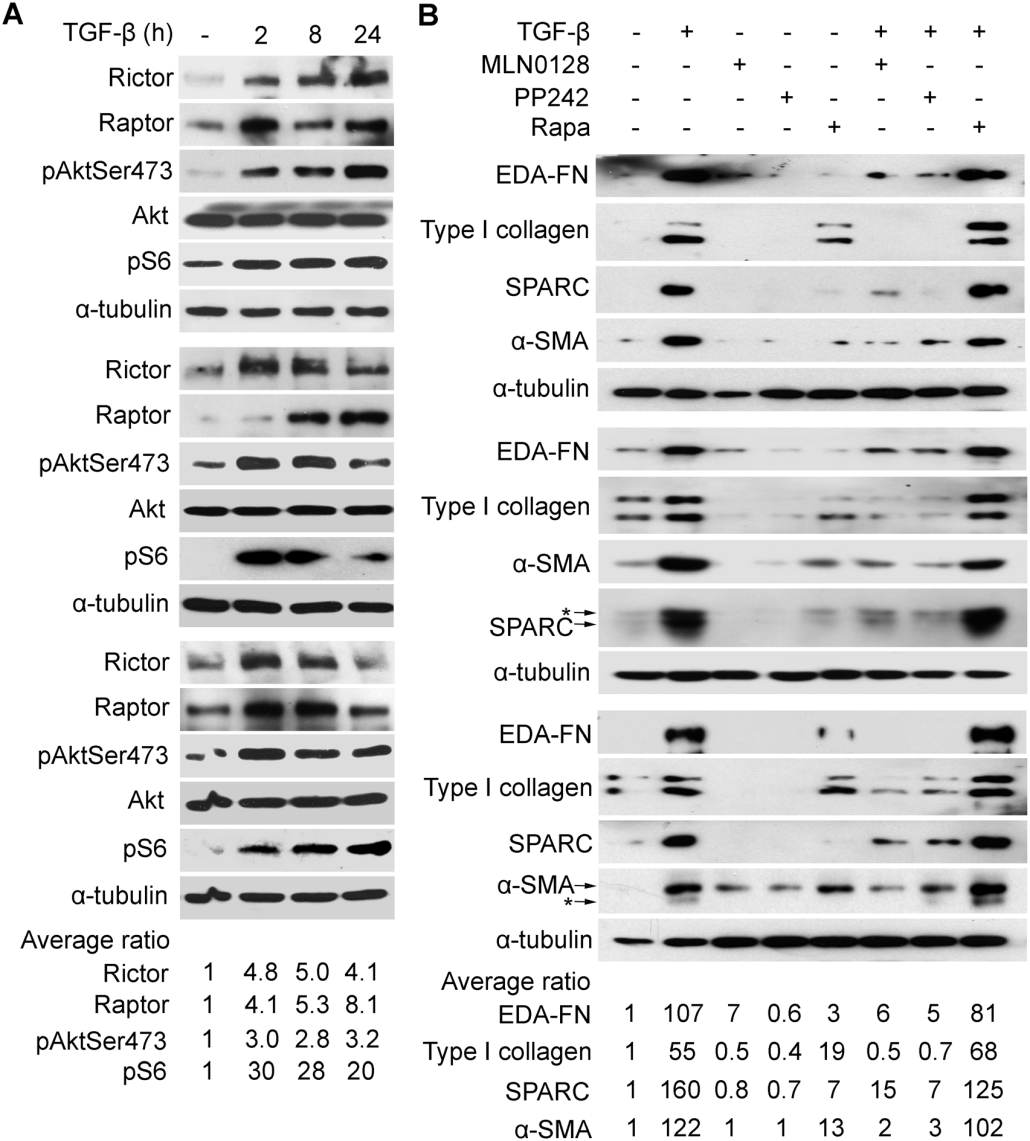
Rictor is a target of TGF-β and the effect of mTOR inhibitors on TGF-β signaling in IPF lung fibroblasts. IPF fibroblasts (<passage 8) isolated from surgical lung biopsy (top panel) or lung transplant patients (middle and lower panels) were serum-starved for 24 hours prior to treatment. In (A) cells were treated with TGF-β (5 ng/ml) for time as shown; (B) cells were treated with TGF-β (5 ng/ml) overnight or left untreated in the presence or absence of indicated inhibitors MLN0128 (0.2 µM), PP242 (2 µM), or rapamycin (Rapa, 0.05 µM), which were added 30 minutes prior to TGF-β. Total cell lysates were prepared and equal amounts of protein were analyzed by Western blot analysis with specific antibodies as indicated. α-tubulin was used as a loading control. Asterisk indicates the carry-over signals between the western blots of α-SMA and SPARC. Band intensity was determined by using Image J software from the NIH. Data was presented as band intensity relative to untreated samples. EDA-FN, extra domain A fibronectin; SPARC, secreted protein acidic and rich in cysteine; α-SMA, α-smooth muscle actin.

**Figure 2:**
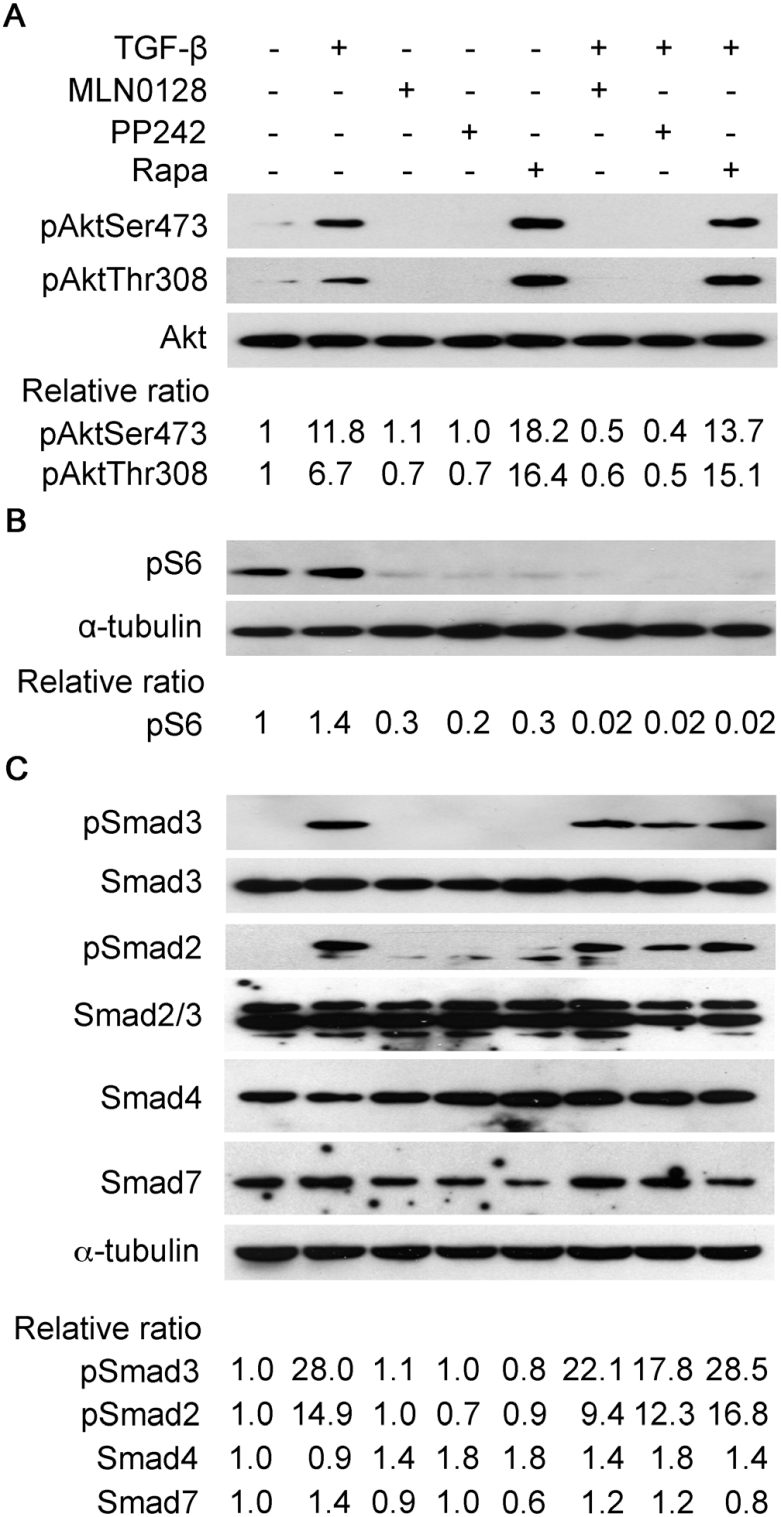
Effect of mTOR inhibitors on TGF-β activation of mTOR and Smad pathways. Serum-deprived IPF fibroblasts were treated with TGF-β for 60 minutes or left untreated in (A), followed by Western blot analysis with anti-phospho Akt (Ser473 or Thr 308) and anti-total Akt antibodies, or in (B) for 6 hours in the presence or absence of indicated inhibitors MLN0128 (0.2 µM), PP242 (2 µM), or rapamycin (0.02 µM), followed by Western blot analysis with anti-phospho-S6 and anti-α-tubulin antibodies. (C) Serum-deprived IPF fibroblasts were treated with or without TGF-β for 15 minutes in the presence or absence of indicated inhibitors followed by Western blot analysis with an anti-phospho-Smad2 or Smad3 antibody. Expression of total Smad-2, 3, 4 and 7 was analyzed by Western blot. Experiment was done on three lines, which are shown in Figure 1; results were similar between the three lines and results from the IPF fibroblasts isolated from surgical lung biopsy are shown here.

Akt is activated by TGF-β and has recently been shown to be a target of mTORC2, so we first examined if TGF-β activates mTORC2 in IPF lung fibroblasts. Rictor is unique to the mTORC2 complex and Raptor to the mTORC1 complex, we looked at the effect of TGF-β on expression of Rictor and/or Raptor- a recent study showed that Rictor is a TGF-β target [23]. We saw that TGF-β induces Rictor in IPF fibroblasts, obtained from patients undergoing surgical lung biopsy (Fig. 1A, upper panel) or lung transplant (Fig. 1A, middle and lower panels). The induction of Rictor coincided temporally with the activation of Akt (phosphorylation at Ser473); levels of Rictor and Akt activation were maximal at 2–8 h in the transplant lines and at 24 h in the biopsy line (Fig. 1A). Raptor was also induced by TGF-β but the induction did not mirror the activation of S6 kinase, a target of mTORC1. Since Rictor is induced by TGF-β in IPF lung fibroblasts and Akt (Ser473) phosphorylation is an mTORC2 target, we surmised that mTORC2 is a downstream target of TGF-β in IPF fibroblasts; therefore, we turned to examine if blocking mTORC2 inhibits TGF-β-mediated induction of an activated fibroblast or myofibrolast phenotype, which is characterized by the induction of alpha smooth muscle actin (α-SMA) and matricellular proteins such as fibronectin, type I collagen, and secreted protein acidic and rich in cysteine (SPARC), also known as osteonectin. However, only inhibitors that target the shared active site of mTORC1 and mTORC2 have been developed; we began our initial studies with the mTORC1 and mTORC2 inhibitor, PP242, an active site mTOR inhibitor, and subsequently advanced to MLN0128, which is structurally similar to PP242 but is approximately 10-fold more potent [24]. In the three IPF fibroblast primary cell lines, we found that PP242 (2.5 µM) and MLN0128 (0.2 µM), but not rapamycin (0.05 µM), suppressed by 50%–80% the basal and TGF-β-inducible expression of type I collagen, the alternatively spliced extra type III domain A fibronectin variant (EDA-FN), α-SMA, and SPARC (Fig. 1B). The selected dose of each inhibitor, i.e., rapamycin, PP242, or MLN0128, mirrors the effective concentration observed in cellular and mouse studies and is in the range of doses being tested in clinical trials [15], [16], [25], [26]. The IC50 of MLN0128 for suppression of stromal proteins by TGF-β is 0.03 µM–0.1 µM (data not shown). Since Akt (Thr308) is a target of PI3K-mediated, PDK1-dependent activation of Akt, we determined if TGF-β also induces phosphorylation of Akt at Thr308 in these cells. We observed that PP242 and MLN0128 blocked TGF-β-induced phosphorylation of Akt at both Ser473 and Thr308, whereas rapamycin caused hyperphosphorylation of Akt (Fig. 2A). All inhibitors blocked the activation of S6 kinase, i.e., phosphorylation, an mTORC1-dependent target (Fig. 2B). Since the canonical TGF-β pathway involves activation of Smad proteins, we examined if any of the mTOR inhibitors block TGF-β-dependent phosphorylation of Smads. Activation of Smad2 or Smad3 by TGF-β was not affected by PP242, MLN0128, or rapamycin (Fig. 2C). Also, TGF-β did not affect expression of Smad4 or Smad7 in these cells (Fig. 2C).

**Figure 3:**
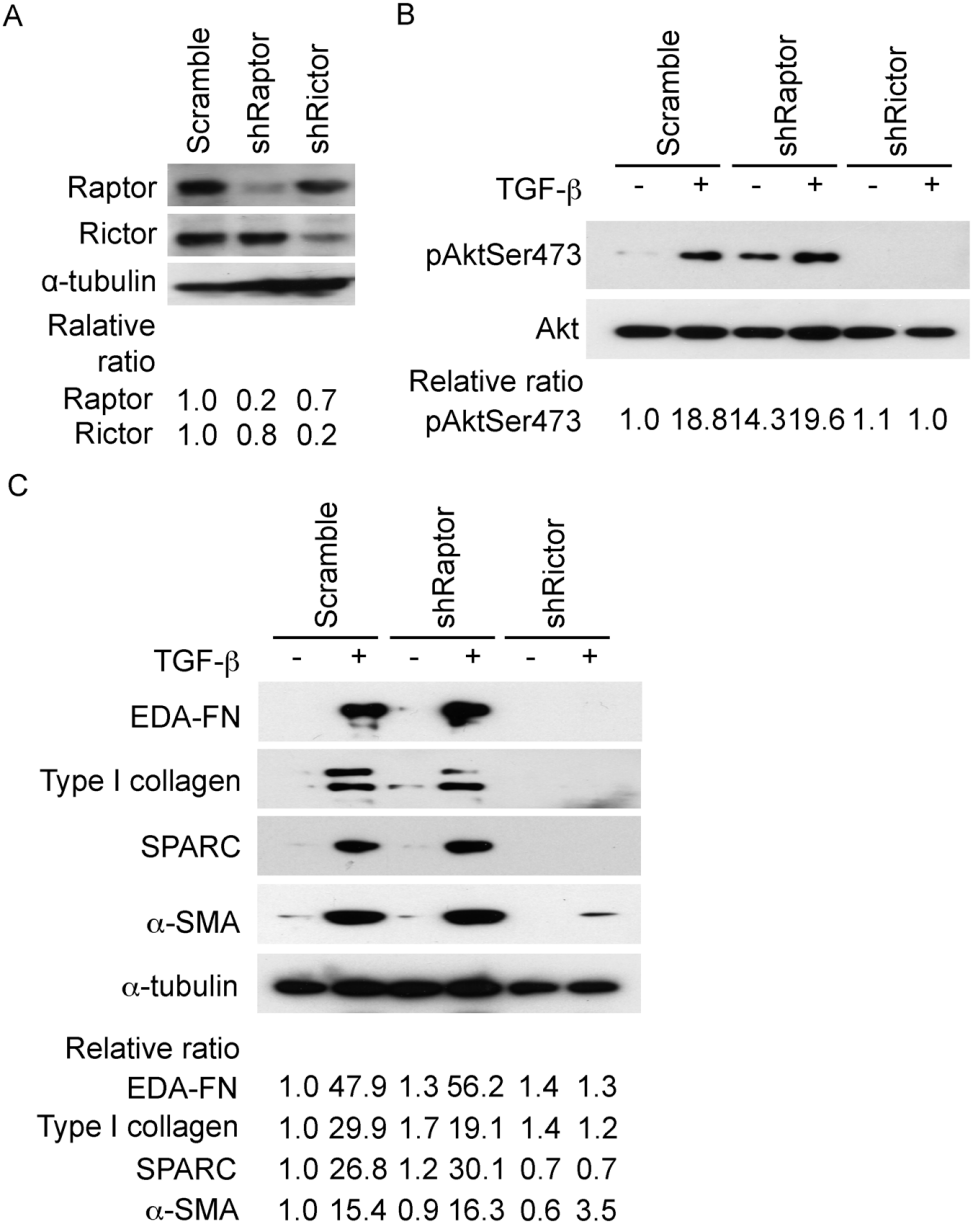
Rictor but not Raptor regulates Akt phosphorylation (Ser473) and the expression of matrix regulatory proteins. In (A) IPF fibroblasts isolated from surgical lung biopsy were infected with lentivirus-derived shRNA against raptor or rictor, or control (scramble) as described in Materials and Methods. Western blot analysis was performed with the indicated antibodies. α-tubulin was used as a loading control. (B) Serum-starved IPF fibroblasts were treated with TGF-β for 60 minutes followed by an analysis of Akt phosphorylation by Western blot analysis. Total Akt was used as a loading control. (C). Serum-deprived IPF fibroblasts were treated overnight with TGF-β followed by analysis of matrix-regulatory proteins by Western blot analysis. α-tubulin was used as a loading control. Experiments with the three IPF lines showed similar results and representative results from the surgical lung biopsy fibroblasts are shown.

In order to confirm mTORC2 as a target of TGF-β, we investigated the effect of depleting Rictor or Raptor by RNA interference. Depletion of Rictor, but not Raptor suppressed TGF-β activation of Akt; interestingly, shRaptor increased the basal activation of Akt, (Fig. 3A), similar to what we had observed with rapamycin (Fig. 2A). Moreover, the downregulation of Rictor, but not Raptor, inhibited the expression of markers of activated fibroblasts (Fig. 3B), similar to our observed inhibitory effect of MLN0128 (Fig. 1B). MLN0128 alone caused a 15%–20% reduction in the viability of IPF lung fibroblasts (Fig. S1).

**Figure 4:**
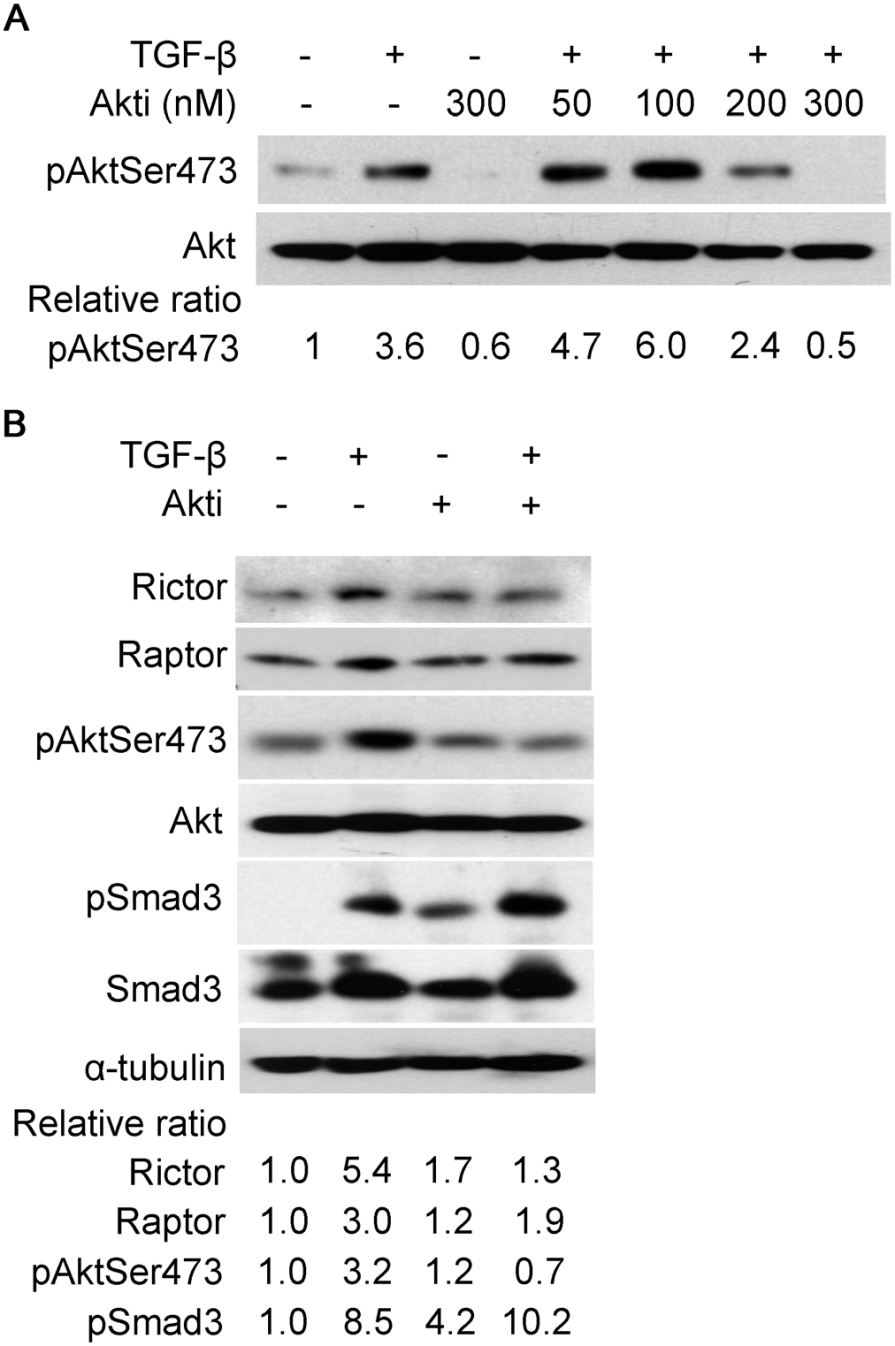
Akt inhibition suppresses induction of Rictor by TGF-β. Serum-starved IPF fibroblasts were pre-treated with Akti (Akt inhibitor VIII/124018) for 30 minutes or left untreated prior to TGF-β (5 ng/ml) treatment for two hours. In (A) cells were pre-treated with Akti at indicated concentration as shown, then followed by TGF-β treatment; (B) cells were pre-treated with Akti at 300 nM prior to TGF-β treatment or left untreated. Total cell lysates were prepared and equal amounts of protein were analyzed by Western blot analysis with specific antibodies as indicated. α-tubulin was used as a loading control.

To ascertain if Rictor induction by TGF-β is mediated by Akt, we applied the specific Akt inhibitor, Akti (Akt inhibitor VIII/124018, Millipore, Billerica, MA). Akti caused a dose-dependent inhibition of Akt activation (Fig. 4A). Also, Akti (300 nM) suppressed Rictor induction by TGF-β; inhibition of Akt, however, did not suppress the induction of Raptor (Fig. 4B).

**Figure 5:**
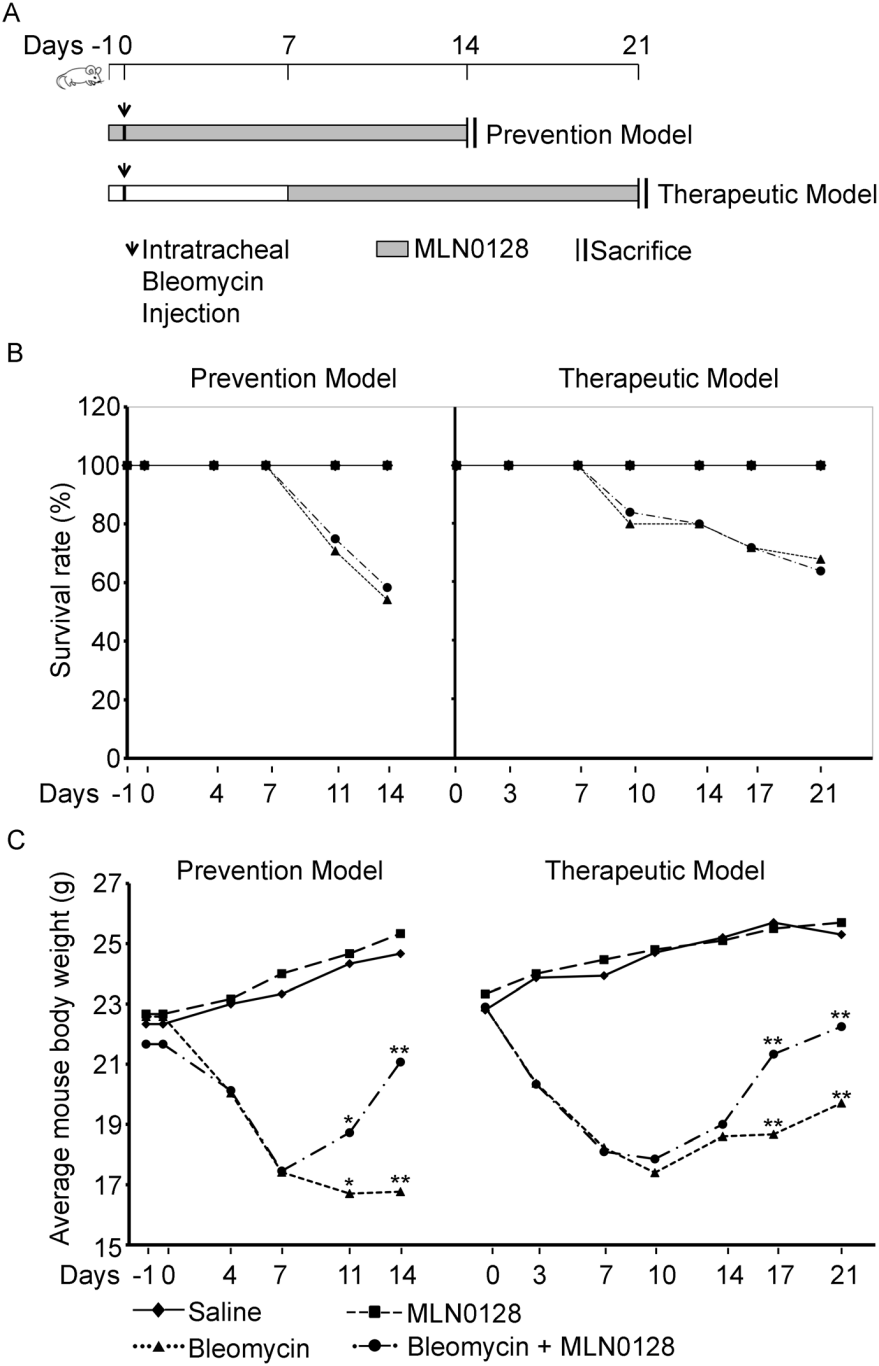
(A) Schematic of bleomycin prevention and therapeutic protocols. (B) Mouse survival rates are from four independent experiments for the prevention model (n = 3 for Saline or MLN groups and n = 6 for Bleo or Bleo + MLN groups) and from five independent experiments for the therapeutic model (n = 3 for Saline or MLN groups, n = 6 for Bleo, and n = 5 for Bleo + MLN groups). (C) Mouse body weights are from bleomycin prevention and therapeutic model experiments (*P<0.05. and **P<0.005) as in (B). Each point represents the mean body weight of mice in the respective treatment group from each experiment.

**Figure 6:**
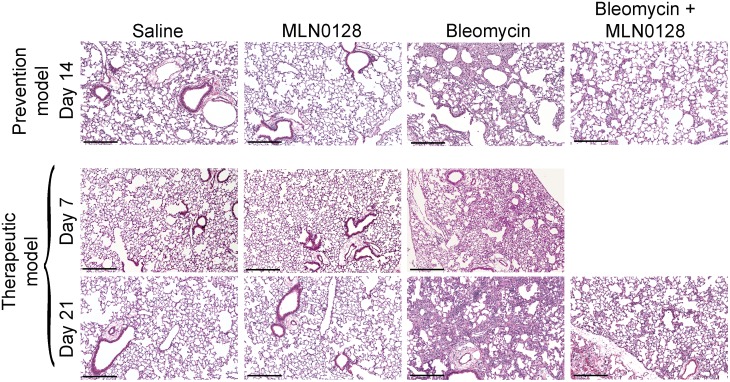
MLN0128 inhibits bleomycin-induced lung fibrosis. Mice were treated according to the schematic shown in Fig. 5A. Mice lungs were harvested at Day 14 (prevention model) or Day 21 (therapeutic model) followed by H&E staining. Scale bar = 100 micron.

**Figure 7:**
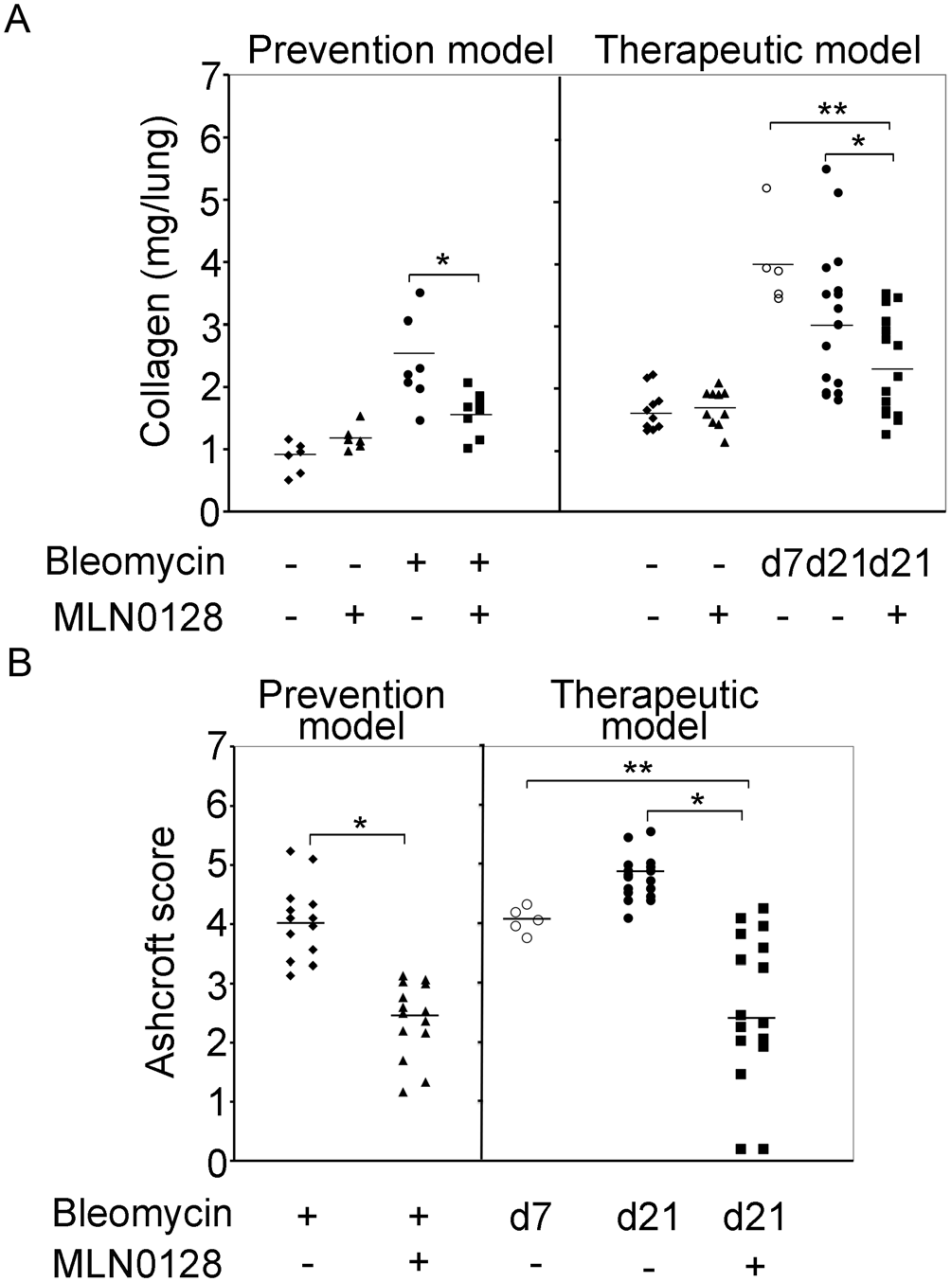
MLN0128 inhibits bleomycin-induced fibrosis. In (A) mice were treated as described in Fig. 5A followed by harvest of the right lung for a Sircoll collagen assay. The horizontal bar represents the mean value of collagen content (mg/lung) for each sample group. *P<0.05. (B) Analysis of Ashcroft score in left lung of mice from (A); *P<0.001. Data shown is combined from four independent prevention model and five independent therapeutic model experiments.

To explore the anti-fibrotic activity of MLN0128 *in vivo* we examined its effect in the murine lung bleomycin model. MLN0128 was administered as part of a prevention strategy, i.e., treatment initiation on Day −1, one day prior to bleomycin insult, or a delayed therapeutic strategy, i.e., treatment starting at Day 7 after bleomycin (Fig. 5A). We chose intraperitoneal injection for MLN0128, even though it is orally administered in clinical trials with cancer patients, because mice ailing from bleomycin treatment did not tolerate oral gavage with the vehicle routinely used to dissolve MLN0128 (15% polyvinylpyrrolidone K30). An MLN0128 dose of 0.75 mg/kg/d was selected based on its efficacy and lack of toxicity in animal murine cancer models [15], [26]. Mice were treated daily (6/7 days) with MLN0128, and sacrificed at Day 14 in the prevention model or at Day 21 in the therapeutic model, respectively. There was no significant difference in mortality in the bleomcyin control versus MLN0128 treatment group (Fig. 5B). However, body weight significantly improved in MLN0128 treatment groups in both the prevention (Day 14) and therapeutic models (Day 21) (Fig. 5C). In both the prevention and therapeutic models, MLN0128 significantly inhibited bleomycin-induced lung fibrosis (Fig. 6) and collagen content (Fig. 7A); also, MLN0128-treated mice had a significantly lower Ashcroft score (Fig. 7B). Moreover, MLN0128 reduced picosirius red staining, another measure of collagen content (Fig. S2 and Text S1). There was no observable lung toxicity with MLN0128 (Fig. S4).

We then examined the effect of MLN0128 in the prevention model on mRNA expression of known TGF-β responsive genes (Fig. S3 and Text S1). There was no significant increase in Type I collagen, Type III collagen, SPARC, or α-SMA at Day 14 after bleomycin administration (Fig. S3). However, bleomycin caused a significant increase in matrix-regulatory genes, plasminogen activator inhibitor 1 (PAI-1), S100A4, also known as fibroblast specific protein-1 (FSP-1) or metastasin1 (MTS1), and FN gene expression, which were all significantly inhibited by MLN0128 (Fig. S3) [27].

**Figure 8:**
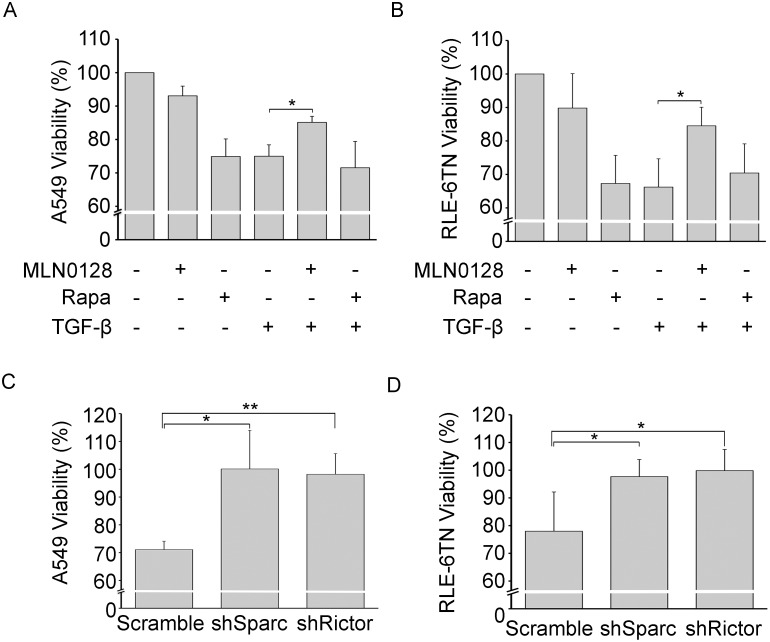
MLN0128 blocks TGF-β-mediated attenuation of lung epithelial cell viability. (**A**) A transwell culture protocol, as described in detail in Materials and Methods, using IPF fibroblasts co-cultured with A549 cells (*P<0.005) or (**B**) RLE-6TN cells (*P<0.001), which was followed by analysis of A549 or RLE-6TN viability by an Alamar Blue assay. (**C**) Downregulation of SPARC or Rictor in A549 (*P<0.05; **P<0.005) or (**D**) RLE-6TN cells (*P<0.05) by RNA interference in TGF-β-treated IPF fibroblasts was followed by an Alamar Blue assay of A549 or RLE-6TN cells. Data is expressed as mean +/− standard deviation from two IPF fibroblast lines (isolated from surgical lung biopsy and lung transplant) in three independent experiments.

In IPF fibroblastic foci, it is generally believed that type II alveolar epithelial cells are damaged by activated fibroblasts. It has previously been shown in a Transwell co-culture system that TGF-β-stimulated fibroblasts impair the viability of lung epithelial cells [28]. We utilized this assay to determine if MLN0128 attenuates the TGF-β-mediated reduction in lung epithelial viability. We saw a 25%–30% reduction in lung epithelial viability of A549 or RLE-6TN cells, which were co-cultured with TGF-β-stimulated IPF lung fibroblasts (Fig. 8A, B). Also, treatment of unstimulated IPF fibroblasts with rapamycin reduced lung epithelial viability in both cell lines and rapamycin did not protect against the reduction in viability by TGF-β (Fig. 8A, B). In contrast, treatment of TGF-β-stimulated IPF fibroblasts with MLN0128 blocked the TGF-β-mediated reduction in epithelial viability (Fig. 8A, B). Using the Transwell co-culture assay, a recent paper by Shibata, et al, showed that the SPARC secreted by TGF-β-treated normal lung fibroblasts impairs lung epithelial viability [29]. We extended this analysis to IPF fibroblasts, where we depleted SPARC by RNA interference [12]. Downregulation of SPARC almost completely restored A549 or RLE-6TN viability following the TGF-β treatment of IPF fibroblasts (Fig. 8C, D). Since the mTORC2 pathway likely regulates SPARC expression in IPF fibroblasts (Fig. 1B and 3), we examined the effect of downregulation of Rictor in TGF-β-treated IPF lung fibroblasts on lung epithelial viability. Similar to turning down SPARC, the downregulation of Rictor almost completely restored A549 or RLE-6TN viability (Fig. 8C, D).

**Figure 9:**
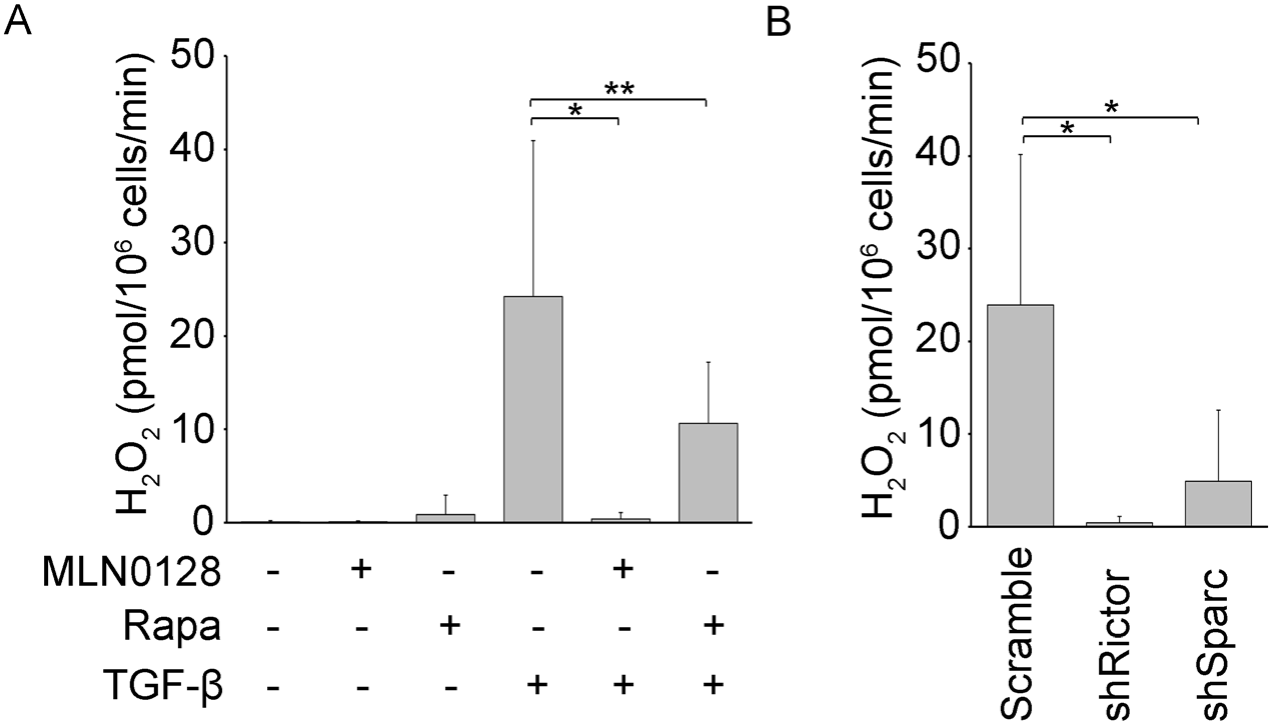
H_2_O_2_ release from IPF fibroblasts is mediated by SPARC and mTORC2. (**A**) IPF fibroblasts were treated for 16 h with TGF-β alone or in combination with MLN0128 (0.2 µM) or rapamycin (0.05 µM) followed by measurement of H_2_O*_2_* (*P<0.05, **P>0.05)_,_ as described in detail in Materials and Methods. (**B**) SPARC or Rictor was downregulated by RNA interference in TGF-β-treated IPF fibroblasts followed by measurement of H_2_O*_2_*; *P<0.05_._ Data is expressed as mean +/− standard deviation from same two fibroblast lines as in Figure 8, in three independent experiments.

In the study by Shibata, et al, the authors contend that a SPARC-mediated induction of hydrogen peroxide (H_2_O_2_) production by lung fibroblasts impaired lung epithelial viability [29]. Since SPARC is a target of the mTORC2 pathway, we examined a role for mTORC2 by adding MLN0128 or by Rictor downregulation in this co-culture system. We found that MLN0128 or Rictor downregulation causes a 90% and 80% reduction in H_2_O_2_ release respectively (P<0.05) (Fig. 9A). Also, the downregulation of SPARC suppressed H_2_O_2_ production by 95% (P<0.05); rapamycin decreased H_2_O_2_ production by 40% (P>0.05) (Fig. 9B).

## Discussion

The mTOR pathway has a broad regulatory role in metabolism, cell growth, tumorigenesis, and development. However, until recently, the majority of research and published studies have focused on the rapamycin-sensitive mTORC1 component of the pathway. Once it was revealed that Akt is activated by mTORC2, there have been several recent studies defining functions of mTORC2, which are distinct from mTORC1 [6]. For example, mTORC2 regulates growth factor dependent signaling, glycolysis, and epithelial-mesenchymal transition (EMT) [6]; most recently, a study by Goncharov, et al, showed that mTORC2 regulates the glycolytic pathway and mediates increased proliferation and survival of pulmonary artery vascular smooth muscle cells in Idiopathic Pulmonary Arterial Hypertension (IPAH) [30]. Also, evidence is emerging for both transcriptional and translational regulation of Rictor expression. For example, a study showed that Forkhead box (FoxO) transcription factors induce Rictor expression during oxidative or nutrient stress [31], [32]. Also, recent study showed that Rictor is upregulated during S phase of the cell cycle, leading to mTORC2 activation, which is necessary for accurate cell cycle progression [33]. A study by Serrrano, I., et al, showed that TGF-β induces Rictor in cancer cells, that was accompanied by formation of an ILK/Rictor complex, which promoted migration and EMT in mammary cancer cells [23]. Interestingly, the late, but not early (up to 2 h), phase of Akt activation (>24 h) was required for EMT. Moreover, downregulation of the MicroRNAs MiR-424 and MiR503 was shown to upregulate Rictor, which promotes colon cancer progression [34]. In the study here, we found that TGF-β induces Rictor in IPF fibroblasts, and its induction coincides with Akt activation. Our results suggest that Rictor upregulation leads to an mTORC2-dependent sustained activation of Akt in IPF fibroblasts. It is possible that this sustained activation is required for regulation of the activated fibroblast, ie, myofibroblast, phenotype.

We targeted mTORC2-dependent activation of Akt with MLN0128, an active site mTOR inhibitor. Other downstream targets of mTORC2 include AGC kinases, such as PKC-δ, which is downstream of lysophosphatidic acid receptor (LPA)-mediated activation of the G protein, Gα_12_ [35]. LPA appears to play a significant role in lung fibrosis, in part through its induction of fibroblast migration [36]. However, we did not see activation of PKC-δ by TGF-β in IPF lung fibroblasts, suggesting a more prominent role possibly for inhibition of Akt by active site mTOR inhibitors, not PKC-δ, in the inhibition of fibroblast activation and lung fibrosis.

Interestingly, we observed hyperactivation of Akt with rapamycin- other studies have also found that blocking mTORC1 alone with agents like rapamycin or everolimus can lead to undesirable activation of mTORC2 [37]. This may be an underlying cause why everolimus failed in a clinical trial of IPF patients; also, it may be that activation of mTORC2 by rapamycin or everolimus is involved in the pathogenesis of interstitial pneumonitis, which has been observed in 10%–15% of patients treated with these agents [38]. Finally, active site mTOR inhibitors, through targeting the ATP binding motif in mTOR, are also more active in blocking mTORC1 than rapamycin, which is an allosteric partial inhibitor of mTORC1 [39].

Our data from cultured IPF fibroblasts demonstrate the superiority of active site mTOR inhibitors over rapamycin in suppression of expression of pro-fibrotic matrix regulatory proteins, such as type I collagen, EDA-FN, and SPARC, all of which are targets of TGF-β. We show here that the dual inhibitor MLN0128 significantly inhibits fibrosis in a prevention and therapeutic murine model of bleomycin-induced lung fibrosis. It is arguable whether administration of an inhibitor, such as MLN0128, remotely from bleomycin injury is in fact a “therapeutic” model, but it is administered after the peak of the inflammatory and injury phase and therefore targets the fibrotic phase of repair. A study by Peng, R. et al also suggests that the bleomycin therapeutic model may be a more clinically relevant model of IPF than the prevention model [40]. We did not observe any evidence of lung or systemic toxicity of MLN0128 at the dose of 0.75 mg/kg/d IP, a dose that yields serum levels analogous to those seen in the higher dose ranges currently being tested in Phase I and Phase II cancer clinical trials. This dose was also well tolerated in a murine tuberous sclerosis model, but there was significant weight loss at a higher dose of MLN0128 (1 mg/kg/d) [26]. Identifying potential biomarkers of targeted inhibition by MLN0128 will be important for designing clinical trials in pulmonary fibrosis patients- PAI-1, FN, and S100A4 are potential biomarkers since they are inhibited by MLN0128 in the bleomycin model (Figure S3). Investigating the inhibition of Akt activation in peripheral blood and bronchoalveolar lavage cells (BAL) could be a logical readout of mTORC2 inhibition. In fact, a new Phase I study of a specific PI3K inhibitor in IPF by GlaxoSmithKline proposes to look at Akt activation in platelet-rich plasma and BAL cells as a biomarker of drug activity (ClinicalTrials.gov-NCT1725139).

There is no well-described *in vitro* mimic of the epithelial-fibroblastic crosstalk, which occurs in fibroblastic foci in IPF lung and other fibrotic lung diseases. Injury and depletion of the type II AEC likely contributes to the unrelenting process of dysregulated repair and progressive fibrosis in IPF; however, the precise role of the fibroblast in mediating epithelial injury and its loss is incompletely understood. Since secreted matricellular proteins like PAI-1 and SPARC are expressed by fibroblasts in fibroblastic foci, they are in the perfect biological context in IPF lung to influence lung epithelial cell behavior; therefore, we set out to recapitulate epithelial-fibroblast crosstalk using a compartmentalized Transwell system. Surprisingly, rapamycin alone led to a reduction in epithelial viability suggesting that rapamycin causes the fibroblast to secrete a factor(s) that is harmful to lung epithelium (Fig. 8). Since SPARC is downstream of TGF-β-mediated activation of mTORC2 signal transduction, we speculated that mTORC2 and SPARC plays a role in mediating the protective effect of MLN0128; this was especially likely in that Shibata, S., and Ishiyama, J., recently published that fibroblast-derived SPARC causes a loss of lung epithelial cell viability [29]. In accordance with this, we observed that mTORC2 and SPARC regulate A549 or RLE-6TN lung epithelial viability and their production of H_2_O_2_- a similar amount of H_2_O_2_ was shown to damage small airway lung epithelia using the same Transwell model system [29]. These data suggest a possible *in vivo* correlation in IPF: TGF-β induces SPARC production through mTORC2 and Akt activation in IPF fibroblasts, which then activates H_2_O_2_ production by the fibroblasts, leading to a loss of viability of neighboring type II alveolar epithelial cells.

The failure of multiple clinical trials in IPF of several therapeutic agents has been disheartening; however, two recent trails showed that pirfenidone and nintedanib appeared to slow disease progression in IPF [41], [42]. We present an argument for further investigation of the active site mTOR inhibitors, like MLN0128 in IPF based on its pleiotropic effects, which include the inhibition of production of pro-fibrotic proteins by IPF fibroblasts, efficacy in the murine bleomcyin model, and protection of lung epithelium. However, the safety profile of an antiproliferative agent like MLN0128 needs to be carefully examined in the IPF population. An obvious question and concern is whether active site mTOR inhibitors will cause interstitial pneumonitis in humans which has been observed with mTORC1 inhibitors such as rapamycin or everolimus. Even though rapamycin-mediated activation of Akt and mTORC2 may be the culprit, lung toxicity may be due to mTORC1 inhibition, which is a target of both rapamycin and active site mTOR inhibitors. Ideally, an active site mTOR inhibitor or another agent in clinical trials for IPF will not only delay physiologic evidence of disease progression but will also be disease modifying.

## Supporting Information

Figure S1 **Effect of MLN0128 on viability of IPF fibroblasts.** Serum-starved IPF fibroblasts were treated with TGF-β (5 ng/ml) for overnight or left untreated in the presence or absence of MLN0128 (0.2 µM), followed by an Alamar Blue assay. The results from untreated or TGF-β treated samples are set as the maximal growth (100%), and the effects of MLN0128 are presented as relative percentage change. Results are presented as mean +/− standard deviation from three IPF fibroblast lines (*P<0.001).
(TIF)

Figure S2 **MLN0128 inhibits collagen expression in the bleomycin lung therapeutic model.** H&E and Picrosirus Red staining of formalin fixed paraffin-embedded lung section harvested at Day 21 after the treatments is shown. The quantification of bleomycin vs. bleomycin + MLN0128 yielded the color difference of 9.05% vs. 3.37%, respectively from an analysis by Image J software from the NIH. Scale bar = 100 micron.
(TIF)

Figure S3 **Effect of MLN0128 on gene expression in the bleomycin model.** Expression of several matrix-regulatory genes was examined by harvesting RNA from right lung at Day 14 of bleomycin prevention model followed by analysis of genes indicated by reverse transcriptase reaction and quantitative PCR (n = 4–6 mice per group; *P<0.05, **P<0.005). Results are presented as mean +/− standard deviation, and are combined from four independent experiments. α-SMA, α-smooth muscle actin; COL1a, collagen Ia; COL3a, collagen IIIa.
(TIF)

Figure S4 **Effect of MLNO128 on mouse lung.** H&E staining of formalin fixed paraffin embedded lung section harvested at Day 7 and 14 after the treatments in the prevention model was shown. Scale bar = 100 micron.
(TIF)

Text S1 **Supporting Methods.**
(DOCX)
