# Supplementary figures and images for: A Critical Role for the mTORC2 Pathway in Lung Fibrosis
Source: PLoS One. 2014 Aug 27;9(8):e106155. doi: 10.1371/journal.pone.0106155 (PMC4146613; doi:10.1371/journal.pone.0106155)

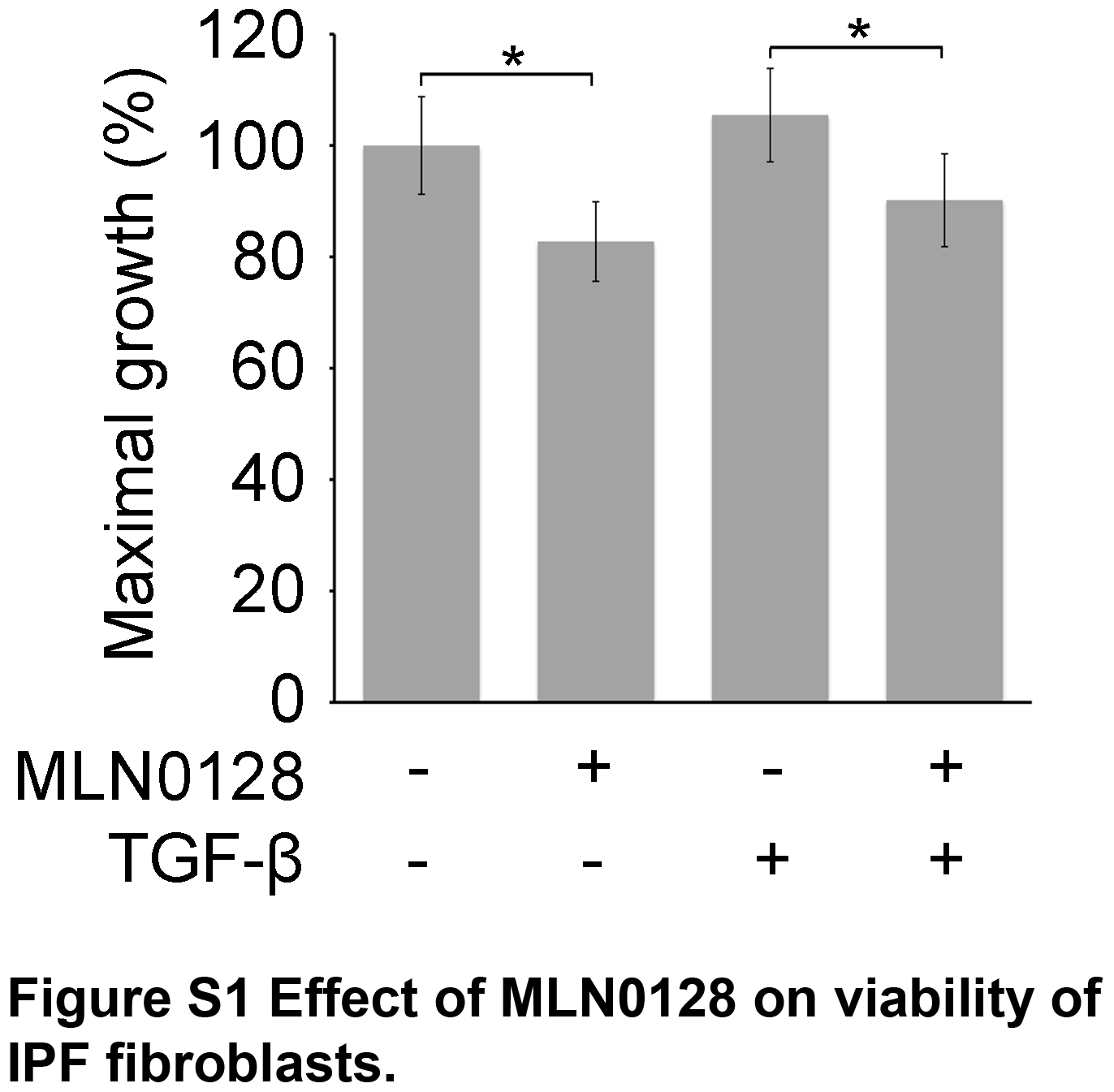

Supplement: Figure S1 — Effect of MLN0128 on viability of IPF fibroblasts. Serum-starved IPF fibroblasts were treated with TGF-β (5 ng/ml) for overnight or left untreated in the presence or absence of MLN0128 (0.2 µM), followed by an Alamar Blue assay. The results from untreated or TGF-β treated samples are set as the maximal growth (100%), and the effects of MLN0128 are presented as relative percentage change. Results are presented as mean +/− standard deviation from three IPF fibroblast lines (*P<0.001). (TIF) [file pone.0106155.s001.tif]

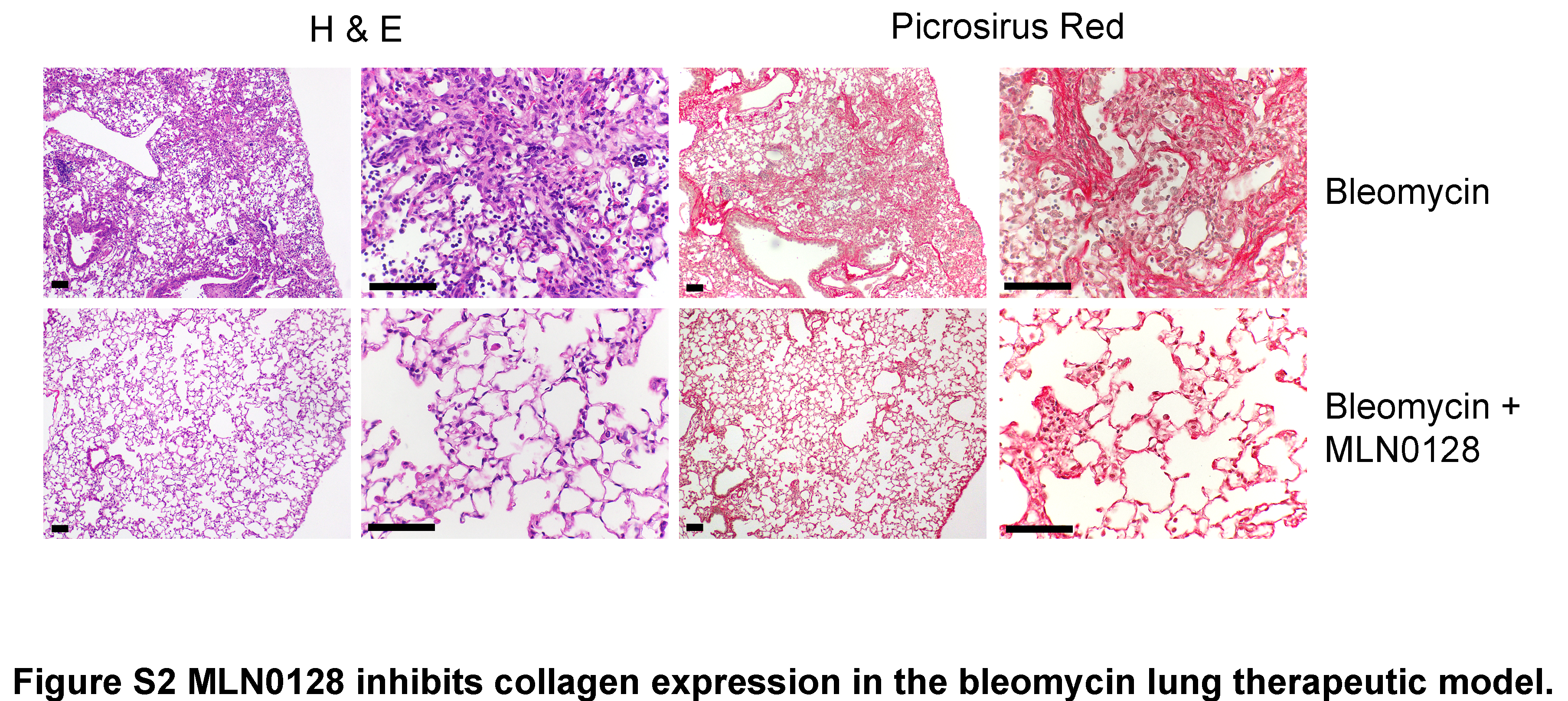

Supplement: Figure S2 — MLN0128 inhibits collagen expression in the bleomycin lung therapeutic model. H&E and Picrosirus Red staining of formalin fixed paraffin-embedded lung section harvested at Day 21 after the treatments is shown. The quantification of bleomycin vs. bleomycin + MLN0128 yielded the color difference of 9.05% vs. 3.37%, respectively from an analysis by Image J software from the NIH. Scale bar = 100 micron. (TIF) [file pone.0106155.s002.tif]

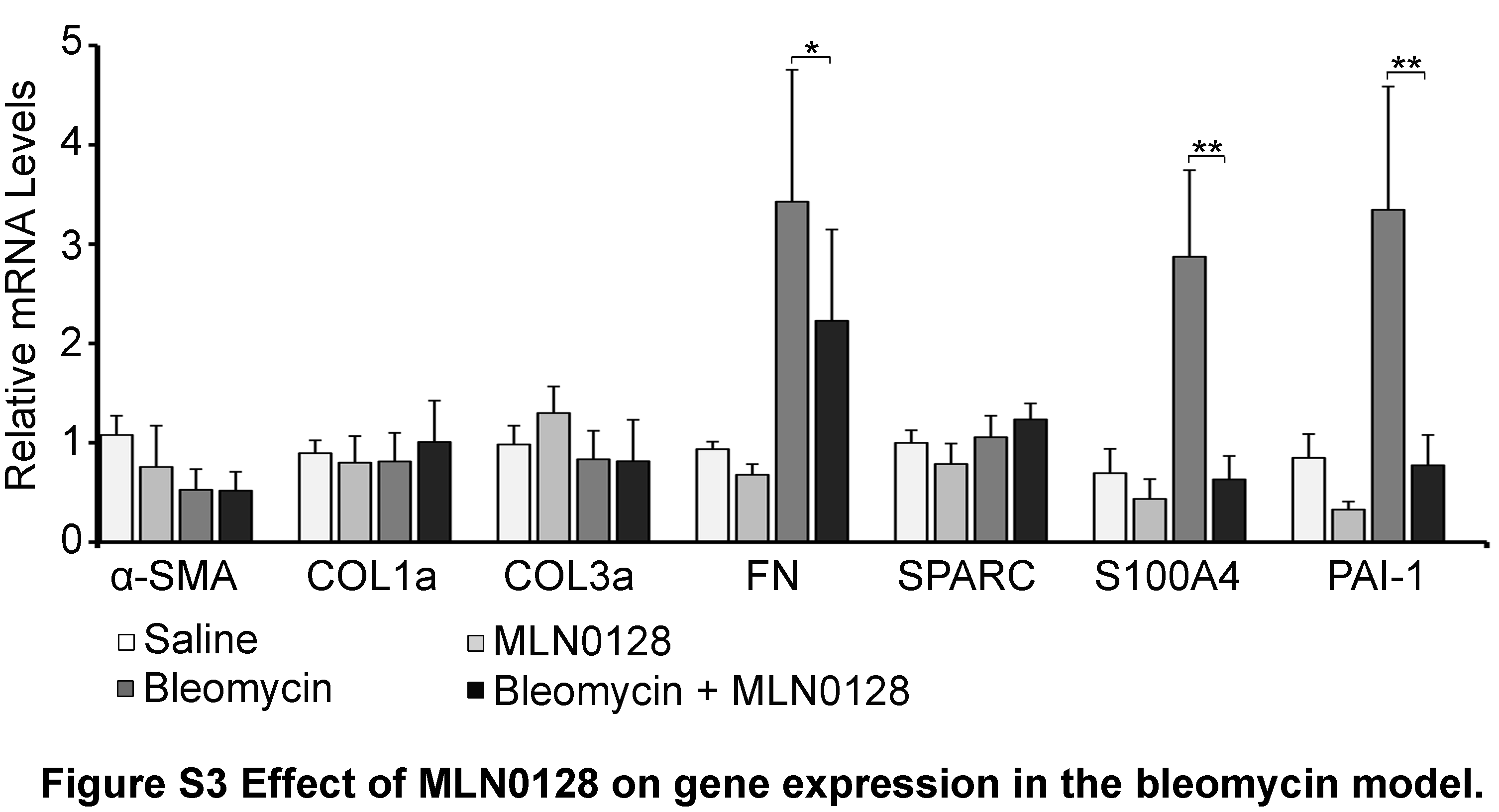

Supplement: Figure S3 — Effect of MLN0128 on gene expression in the bleomycin model. Expression of several matrix-regulatory genes was examined by harvesting RNA from right lung at Day 14 of bleomycin prevention model followed by analysis of genes indicated by reverse transcriptase reaction and quantitative PCR (n = 4–6 mice per group; *P<0.05, **P<0.005). Results are presented as mean +/− standard deviation, and are combined from four independent experiments. α-SMA, α-smooth muscle actin; COL1a, collagen Ia; COL3a, collagen IIIa. (TIF) [file pone.0106155.s003.tif]

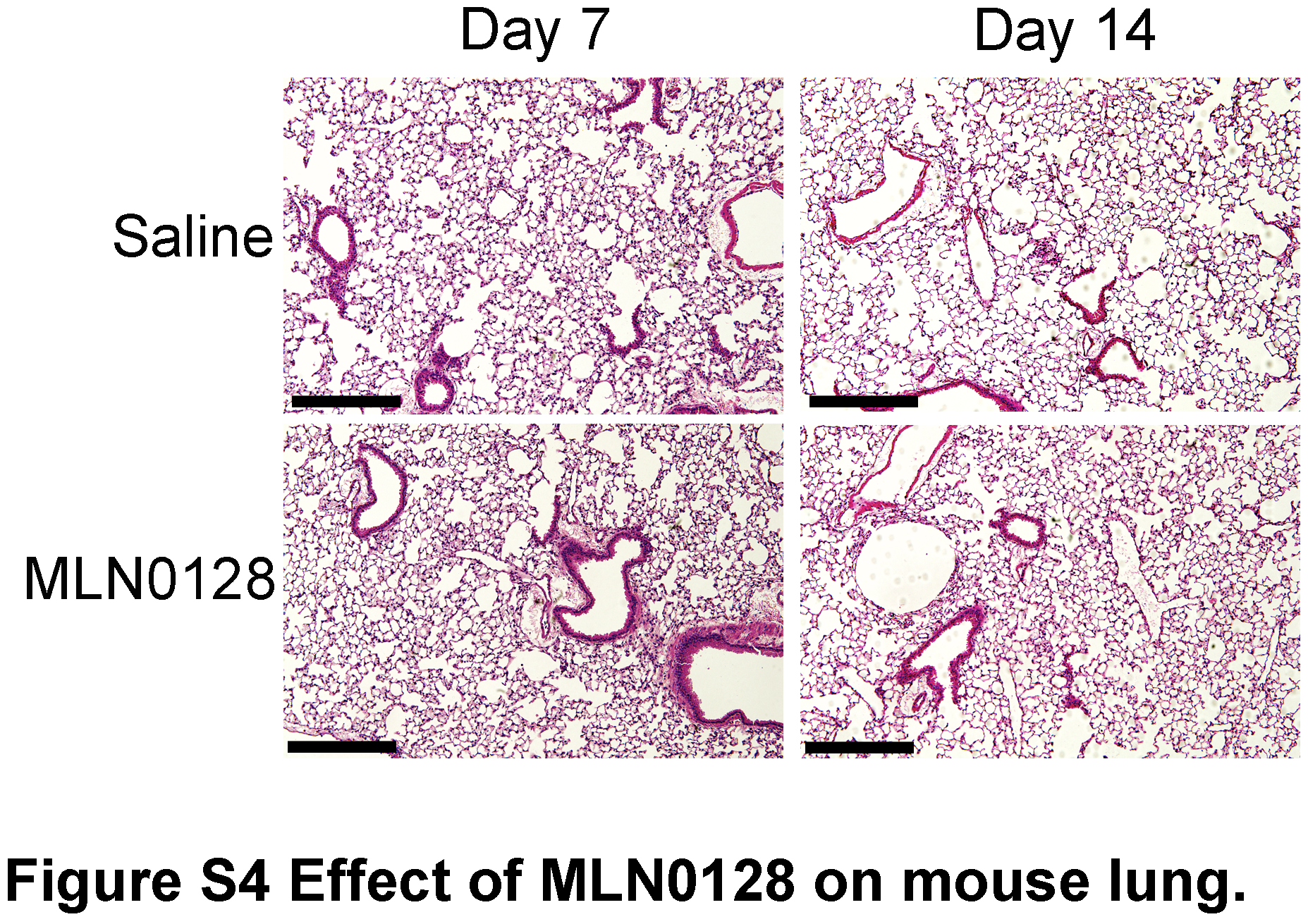

Supplement: Figure S4 — Effect of MLNO128 on mouse lung. H&E staining of formalin fixed paraffin embedded lung section harvested at Day 7 and 14 after the treatments in the prevention model was shown. Scale bar = 100 micron. (TIF) [file pone.0106155.s004.tif]
