# Supplementary material for: A Critical Role for the mTORC2 Pathway in Lung Fibrosis
Source: PLoS One. 2014 Aug 27;9(8):e106155. doi: 10.1371/journal.pone.0106155 (PMC4146613; doi:10.1371/journal.pone.0106155)
Supplement: Text S1 — Supporting Methods. (DOCX) [file pone.0106155.s005.docx]

**Text S1**

**Supporting Methods:**

*Analysis of gene expression-* The gene expression analysis was described previously (S1). RNA was extracted from the right mouse lungs using RNeasy Plus Mini Kit from Qiagen (Valencia, CA), and cDNA converted from 5 µg of total RNA was obtained using a SuperScript First Strand Synthesis System for RT/PCR kit (Invitrogen Corporation, Carlsbad, CA). To control for genomic DNA contamination, additional RNA samples were processed without reverse transcriptase. The RT product equivalent to 25 ng of total RNA was then added to a real time qPCR reaction together with the Dynamo SYBR Green qPCR Kit (Finnzymes, Espoo, Finland) according to the manufacturer’s protocol with the following mouse primers (forward and reverse); SPARC, (5’-AGGTGTGTGAGCTGCACGAGA-3’ and 5’-GAAGTGGCAGGAAGAGTCGAA-3’); α-smooth muscle actin (α-SMA); (5’-TGCTGA CAGAGGCACCACTGAA-3’ and 5’-CAGTTGTACGTCCAGAGGCATAG-3’); Collagen Ia (5’-CCTCAGGGTATTGCTGGACAAC-3’ and 5’-CAGAAGGACCTTGTTTGCCAGG-3’); Fibronectin (5’-GAAGTCGCAAGGAAACAAGC-3’ and 5’-GTTGTAGGTGAACGGGAGGA-3’); PAI-1 (5’-GACACCCTCAGCATGTTCATC-3’ and 5’-AGGGTTGCACTAAACATGTCA-3’); S100A4 (5’-GGAGCTGCCTAGCTTCCTG-3’ and 5’-GCTGTCCAAGTTGCTCATCA-3’); glyceraldehyde 3-phosphate dehydrogenase (GAPDH), (5’-TTGTCTCCTGCGACTTCA-3’ and 5’-CACCACCCTGTTGCTGTA-3’). Real Time qPCR was performed in strip tubes using a StepOne PCR System (Applied Biosystems, Foster City, CA) according to the manufacture’s instructions. The specificity of amplified products was suggested by a melting curve resulting in only one peak. This was further confirmed by agarose gel electrophoresis of the PCR products visualized under ethidium bromide-UV illumination. Target amplifications were compared to the reference amplifications (GAPDH) in the same experiment for each RT product tested. All reactions were carried out in duplicate and the threshold cycle (Ct) values were determined by automated threshold analysis with the StepOne Software. The final results were presented as relative fold-change in target gene expression compared to reference based on comparative or ΔΔCt method. The efficiency of each primer pair was determined by the qPCR procedure from standard dilutions of cDNA (equivalent to 10 pg-10 ng of total RNA in RT reaction).

*Picrosirius staining-* Sections were stained with 0.1% Picrosirius solution (w/v of Direct Red 80 from Sigma in saturated aqueous picric acid) for 1–2 h, washed for 1 min in 0.01 N HCl, then counterstained with hematoxylin (S2).

**References**

S1. Chang W, Wei K, Jacobs SS, Upadhyay D, Weill D, Rosen GD. SPARC suppresses apoptosis of idiopathic pulmonary fibrosis fibroblasts through constitutive activation of β-catenin. The Journal of Biological Chemistry 2010; 285: 8196-8206.

S2. Junqueira LC, Bignolas G, Brentani RR. Picrosirius staining plus polarization microscopy, a specific method for collagen detection in tissue sections. Histochem J 1979; 11: 447-455.
